# The global impact of imiglucerase therapy in children with Gaucher disease types 1 and 3: a real-world analysis from the International Collaborative Gaucher Group Gaucher Registry

**DOI:** 10.1186/s13023-026-04282-w

**Published:** 2026-03-11

**Authors:** Pramod K. Mistry, Jenny L. Carwile, Thomas Andrew Burrow, Azza Abdel Gawad Tantawy, Jose Simon Camelo, Jaya Ganesh, Isabela Batsu, Maria Gabriela Perichon, Antonio Oliveira-dos-Santos, Julie L. Batista

**Affiliations:** 1 Yale University School of Medicine, 333 Cedar Street, LMP 1080, New Haven, CT 06520 USA; 2 Sanofi, Cambridge, MA USA; 3 Arkansas Children’s Hospital, University of Arkansas for Medical Sciences, Little Rock, AR USA; 4 Ain Shams University, Cairo, Egypt; 5 Ribeirão Preto Medical School, University of São Paulo, São Paulo, Brazil; 6 Icahn School of Medicine at Mount Sinai, New York, NY USA; 7 Sanofi, Bridgewater, NJ USA; 8 Sanofi, Rio de Janeiro, Brazil

**Keywords:** Gaucher disease type 1, Gaucher disease type 3, Imiglucerase, ICGG Gaucher Registry, Early intervention, Growth failure, Neuronopathic Gaucher disease

## Abstract

**Background:**

Gaucher disease (GD) in children is highly heterogeneous, severe, and particularly devastating in GD type 3 (GD3), which has systemic and neurological involvement. Imiglucerase enzyme replacement therapy is well-established for managing hematovisceral and skeletal manifestations in GD type 1 (GD1). Its long-term impact in GD3 remains underexplored, with evidence limited to small, single-center cohorts and one registry analysis with limited follow-up.

**Methods:**

This longitudinal analysis used data from the International Collaborative Gaucher Group (ICGG) Gaucher Registry as of January 2023. Children diagnosed with GD1 or GD3 who started imiglucerase before age 18 years and had an intact spleen and at least one baseline and follow-up measurement were included. Linear mixed models were used to evaluate long-term treatment response of hemoglobin, platelet count, liver and spleen volumes, and Z-scores for height, weight, body mass index, and total lumbar spine bone mineral density (BMD).

**Results:**

In total, 961 children with GD1 and 236 with GD3 met the inclusion criteria, representing a population with severe disease requiring early imiglucerase therapy at a median age (25^th^, 75^th^ percentile) of 7.8 (4.3, 12.0) years for GD1 and 1.9 (1.4, 3.4) years for GD3. Significant hematological and visceral improvements were observed within the first 1.5 years of treatment (*p* < 0.001 for all parameters), followed by incremental gains during up to two decades. Height Z-scores increased substantially during the first three years of treatment (*p* < 0.001). Total lumbar spine BMD Z-scores improved (*p* < 0.05).

**Conclusions:**

This is the largest, most comprehensive global evaluation of long-term imiglucerase therapy in pediatric GD1 and GD3. Despite complex disease burden in GD3, imiglucerase therapy led to marked hematovisceral disease reversal and meaningful growth improvements, closely paralleling responses in GD1. These findings emphasize the importance of early diagnosis and sustained treatment to optimize long-term outcomes.

**Registration (registry):**

NCT00358943 (registration date: April 1, 1991).

**Clinical trial:**

Not applicable.

**Supplementary information:**

The online version contains supplementary material available at 10.1186/s13023-026-04282-w.

## Introduction

Gaucher disease (GD) is a rare lysosomal storage disorder caused by biallelic mutations in the *GBA1* gene, leading to deficient activity of acid β-glucosidase [1]. This enzymatic defect results in accumulation of glucosylceramide (GL-1/Gb1) and glucosylsphingosine (lyso-GL-1/lyso-Gb1) within lysosomes, primarily affecting macrophages [1]. In neuronopathic forms—GD type 2 (GD2) and type 3 (GD3)—this pathological accumulation extends to neurons and glial cells, contributing to neuroinflammation and progressive neurodegeneration [1, 2].

GD is broadly classified into three phenotypes based on neurological involvement [3]. GD1, the non-neuronopathic form, is characterized by hepatosplenomegaly, anemia, thrombocytopenia, skeletal complications, and childhood growth failure, with an absence of early-onset central nervous system (CNS) involvement. However, patients with GD1 are at increased risk for Parkinson’s disease and Lewy body dementia later in life [1]. GD2 is the acute neuronopathic form characterized by rapid neurodegeneration and early childhood mortality. GD3, the chronic neuronopathic variant, presents with progressive neurological impairment including horizontal supranuclear gaze palsy, cognitive deficits, and motor dysfunction, as well as profound hematovisceral disease, which is often the most prominent and devastating aspect [3–6]. The disease appears highly inflammatory, with florid systemic inflammation that not only drives massive hepatosplenomegaly, anemia, thrombocytopenia, and skeletal complications but may also have detrimental effects on the brain, potentially exacerbating neurodegeneration [3–6].

Imiglucerase enzyme replacement therapy (ERT) is well-established as a safe and effective treatment for hematovisceral, skeletal, and growth abnormalities in GD1 [7–9]. In many but not all regions, it is also approved for GD3 to manage non-neurological manifestations. While imiglucerase is generally considered unable to cross the blood-brain barrier, its full impact on neuroinflammation and peripheral disease progression in GD3 remains incompletely understood [10, 11].

Although the effectiveness of imiglucerase therapy for GD1 is well-supported by extensive real-world data, including the International Collaborative Gaucher Group (ICGG) Gaucher Registry [9], long-term data on GD3 remain scarce. Previous studies have been limited to small single-center cohorts and one registry analysis with limited follow-up [10, 12–17]. Additionally, the global variability in GD genotypes and phenotypic severity has been largely overlooked, as most prior research has focused on European and North American populations, where the predominant p.Asn409Ser (legacy nomenclature: N370S) genotype results in a milder phenotype. GD3 is more prevalent than GD1 in the Middle East, North Africa, Southeast Asia, and Latin America, where more severe genotypes predominate [5, 12, 13].

GD3 remains significantly underrecognized, underserved and underrepresented in research and clinical guidelines, with limited real-world data guiding treatment decisions. This disparity underscores the need for large-scale, internationally representative studies to inform evidence-based treatment strategies and improve global health equity for this underserved population. To address this critical gap, we conducted the most comprehensive global analysis to date of the long-term outcomes of imiglucerase therapy in children with GD1 and GD3 over nearly two decades in a diverse international cohort, offering an unparalleled look at treatment efficacy across genetically and geographically distinct patient populations.

## Methods

### Study population

Initiated in 1991, the ICGG Gaucher Registry (NCT00358943) is a voluntary, observational, longitudinal, international, clinical database of patients with GD, regardless of treatment status or treatment type. The Registry is governed by a collaborative group of international physician experts in GD and sponsored by Sanofi. Currently, more than 6000 patients in 64 countries are enrolled [8]. Site personnel abstract data from medical records using standardized case report forms and enter it into a central Registry database. Data collection and analysis are conducted according to the principles of the Declaration of Helsinki. Written informed consent is obtained from all patients or legal guardians and verified by study monitors.

We analyzed data downloaded from the Registry database in January 2023, applying the following eligibility criteria: a physician-confirmed diagnosis of GD1 or GD3 and initiation of imiglucerase (Cerezyme, Sanofi, Cambridge, MA) as the first primary GD treatment before 18 years of age. In this study, “imiglucerase” encompasses both imiglucerase and its precursor, alglucerase (Ceredase, Sanofi, Cambridge, MA), for which therapeutic equivalence has been demonstrated [18]. We restricted the analysis to patients with a baseline and at least one follow-up measurement for at least one clinical parameter. Children who were splenectomized or concurrently treated with another GD therapy at the time of imiglucerase initiation were excluded.

### Clinical parameters

The following clinical parameters were assessed: hemoglobin concentration, platelet count, liver and spleen volumes (derived from MRI, CT or ultrasound [19]), height, weight, body mass index (BMI), and total lumbar spine bone mineral density (BMD). Liver and spleen volumes were expressed as multiples of normal (MN) organ size, defined as 2.5% and 0.2% of body weight in kilograms, respectively [7]. Z-scores for height-for-age, weight-for-age, and BMI-for-age were calculated using the 2000 CDC reference population [20] for individuals aged 20 years and younger. Total lumbar spine BMD was measured using dual-energy X-ray absorptiometry (DXA), and Z-scores were reported by individual study sites.

Long-term therapeutic goal thresholds for patients with GD receiving ERT are detailed in the Fig. 4 legend [21, 22]. To maintain data integrity, values falling outside plausible physiological ranges were excluded, including hemoglobin concentrations < 2 or > 20g/dL; platelet counts < 5 or > 2,000 × 10^3^/mm^3^; liver volumes < 0.5 or > 20 MN; spleen volumes > 100 MN; heights < 20 or > 200 cm; weights < 2 or > 180 kg; total lumbar spine BMD Z-scores < −4 or > 4; and height, weight, and BMI Z-scores outside the range of −10 to +10.

### Statistical analysis

For hematologic, visceral, and growth parameters, entry into the study began at imiglucerase initiation. Baseline measurement was defined as the measurement closest to imiglucerase initiation within 3 months before to 2 weeks after initiation for hematologic parameters, 6 months before to 6 weeks after initiation for organ volumes, and 3 months before to 3 months after initiation for growth measurements. For total lumbar spine BMD Z-score, study entry began 4 years after treatment initiation due to sparse data for children with GD3 at the time of treatment initiation; these patients often start treatment at a young age before DXA is typically performed. Moreover, BMD measurement was a relatively recent addition to the standard of care with the realization that the osteoblastic defect in GD results in low peak bone mass in children [23–26]. The follow-up period began 6 months after treatment initiation for all parameters. We defined the end of follow-up as the time at which fewer than 10 children with GD1 or GD3 were remaining: 23 years for hematologic parameters, 13 years for organ volumes, 17 years for height and weight Z-scores, 15 years for BMI Z-scores, and 19 years for BMD Z-scores. Follow-up could extend past the age of 18 years but ended at a maximum age of 20 years for height, weight, and BMI Z-scores. Patients were censored at the time of splenectomy, initiation of combination therapy, or discontinuation of imiglucerase. If a patient reported permanent imiglucerase discontinuation and later restarted imiglucerase, only the first treatment period was included.

Linear mixed models were used to estimate parameter slopes with 95% confidence intervals (CIs) following imiglucerase initiation for each parameter. Therapeutic response to imiglucerase is typically marked by rapid improvement in the early years of treatment, followed by slower, incremental improvement. We employed a piecewise linear approach to account for this pattern, modelling treatment response with two consecutive slopes per parameter for hematologic, visceral, and growth outcomes. Various models with different cut points were assessed, and the optimal fit was selected based on Akaike Information Criteria (AIC). The final cut points were determined: 1 year for hematologic parameters, 1.5 years for visceral parameters (with similar AIC values for 1- and 2-year cut points), and 3 years for growth parameters. Total lumbar spine BMD Z-scores were analyzed after treatment response stabilized, and this parameter was modelled using a single slope.

Models were adjusted for age at treatment initiation (as a continuous variable), sex (all models except organ volumes), and GD type. An interaction term for phenotype and time was included to estimate separate slopes for patients with GD1 and GD3. For each parameter, likelihood ratio tests with fixed effects for sex were used to compare models with and without interaction terms for sex. Treatment response varied by sex for hemoglobin, platelet count, weight Z-score, and BMI Z-score, and separate slopes are presented for males and females. For all other parameters, treatment response did not differ by sex, and slopes are presented for the combined population.

All models incorporated random effects for the intercept (baseline parameter value), follow-up time, and linear spline terms to account for within-individual correlation over time. An unstructured covariance matrix was applied to accommodate variability in measurement number and timing across patients, and models were estimated using restricted maximum likelihood. Statistical significance was evaluated using a two-sided α threshold of 0.05. Statistical analyses were conducted using SAS version 9.4 (SAS Institute Inc., Cary, NC).

## Results

**Fig. 1:**
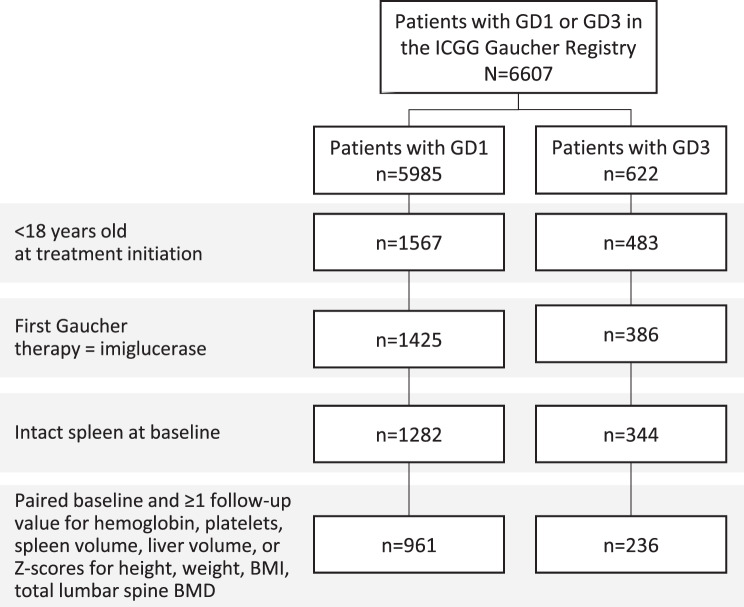
Study population. BMI: Body Mass Index; BMD: Bone Mineral Density. ICGG: International Collaborative Gaucher Group

As of January 6, 2023, the Registry included 6607 patients, with 5985 diagnosed with GD1 and 622 with GD3. Our analysis focused on 961 children with GD1 and 236 with GD3 who met the study’s inclusion criteria (Fig. 1), encompassing 8182 person-years of imiglucerase treatment for GD1 and 1744 person-years for GD3.

Baseline demographics and patient characteristics are shown in Table 1. Age at GD diagnosis ranged from birth to 17.6 years (median 5.3 years) for GD1 and birth to 17.1 years (median 1.6 years) for GD3. Children with GD3 were notably younger on average than those with GD1 at diagnosis and imiglucerase initiation, reflecting the typically severe and debilitating disease course in GD3. Most of the study population was in North America, Europe, and the Middle East, Eurasia, and Africa (EMEA). The majority of children with GD1 carried at least one p.Asn409Ser variant (legacy nomenclature: N370S) (78.4%), whereas most children with GD3 were homozygous for the p.Leu483Pro variant (legacy nomenclature: L444P) (66.5%). Baseline organ volumes and hematologic and growth parameters reflected substantial hematovisceral disease and growth deficit, which were greater among children with GD3 than GD1. Mean (SD) imiglucerase dose at baseline was 45.4 U/kg/2 weeks (30.77) for children with GD1 and 64.0 U/kg/2 weeks (32.85) for children with GD3.

**Table 1:** Baseline demographic and clinical characteristics

Parameters	Patients with GD1 (N = 961)	Patients with GD3 (N = 236)
Region, n (%)		
EMEA	349 (36.3)	136 (57.6)
North America	427 (44.4)	69 (29.2)
LATAM	153 (15.9)	9 (3.8)
JAPAC	32 (3.3)	22 (9.3)
Sex, n (%)	961	236
Male	496 (51.6)	121 (51.3)
Female	465 (48.4)	115 (48.7)
GBA1 genotype*, n (%)	802	212
p.Leu483Pro/p.Leu483Pro	32 (4.0)	141 (66.5)
At least one p.Asn409Ser variant	629 (78.4)	1 (0.5)
All other genotypes	141 (17.6)	70 (33.0)
Age at Gaucher diagnosis (years)	946	231
Mean (SD)	6.2 (4.19)	2.5 (2.92)
Median (25th, 75th)	5.3 (3.0, 8.8)	1.6 (1.1, 2.4)
Age at treatment initiation (years)	961	236
Mean (SD)	8.3 (4.64)	3.3 (3.40)
Median (25th, 75th)	7.8 (4.3, 12.0)	1.9 (1.4, 3.4)
Categories of age at treatment initiation, n (%)	961	236
0-<6 years	365 (38.0)	199 (84.3)
6-<12 years	355 (36.9)	27 (11.4)
12-<18 years	241 (25.1)	10 (4.2)
Age at most recent Registry follow-up (years)	961	236
Mean (SD)	23.1 (9.95)	16.2 (8.92)
Median (25th, 75th)	22.5 (16.2, 30.0)	14.6 (9.4, 23.0)
Hemoglobin (g/dL) at treatment initiation	735	173
Mean (SD)	10.7 (1.71)	9.7 (1.74)
Median (25th, 75th)	10.9 (9.8, 11.8)	9.6 (8.6, 11.1)
Platelet count (x10^3^/mm^3^) at treatment initiation	733	171
Mean (SD)	115 (51)	124 (86)
Median (25th, 75th)	106 (79, 141)	97 (69, 151)
Liver volume (MN) at treatment initiation	340	65
Mean (SD)	1.9 (0.67)	2.3 (0.82)
Median (25th, 75th)	1.8 (1.5, 2.2)	2.2 (1.9, 2.7)
Spleen volume (MN) at treatment initiation	363	76
Mean (SD)	21.1 (14.57)	37.0 (18.32)
Median (25th, 75th)	17.0 (10.6, 28.2)	35.9 (23.5, 47.4)
Height Z-score at treatment initiation	657	166
Mean (SD)	−1.2 (1.39)	−1.9 (1.54)
Median (25th, 75th)	−1.1 (−2.0, −0.2)	−1.8 (−2.6, −0.8)
Weight Z-score at treatment initiation	813	202
Mean (SD)	−0.9 (1.27)	−1.4 (1.50)
Median (25th, 75th)	−0.8 (−1.7, −0.1)	−1.2 (−2.3, −0.6)
Body mass index Z-score at treatment initiation	617	77
Mean (SD)	−0.1 (1.04)	0.3 (1.32)
Median (25th, 75th)	−0.1 (−0.8, 0.5)	0.4 (−0.3, 1.0)
Total lumbar spine BMD Z-score†	312	53
Mean (SD)	−0.6 (1.18)	−0.6 (1.29)
Median (25th, 75th)	−0.6 (−1.4, 0.2)	−0.8 (−1.6, 0.4)
Follow-up time (years), Median (25th, 75^th^ percentile)^#^		
Hematologic parameters	10.5 (4.9, 16.3)	9.4 (4.0, 15.1)
Organ volumes	7.5 (3.5, 11.4)	7.5 (4.2, 11.1)
Height, weight, and BMI	7.1 (3.8, 10.8)	9.4 (4.4, 13.5)
Total lumbar spine BMD	9.7 (6.4, 12.8)	9.9 (5.4, 13.8)
Total measurements per patient, Median (25th, 75^th^ percentile)^#^		
Hemoglobin	17.0 (10.0, 27.0)	15.0 (7.0, 28.0)
Platelet count	17.0 (10.0, 26.0)	15.0 (7.0, 26.0)
Liver volume	6.0 (4.0, 9.0)	7.0 (4.0, 11.0)
Spleen volume	6.0 (4.0, 9.0)	7.0 (4.0, 10.5)
Height	10.0 (6.0, 17.0)	13.0 (6.0, 22.0)
Weight	11.0 (7.0, 19.0)	13.0 (6.0, 22.0)
BMI	11.0 (7.0, 17.0)	14.0 (8.0, 20.0)
Total lumbar spine BMD	3.0 (2.0, 4.0)	3.0 (2.0, 4.0)
Baseline imiglucerase dose (U/kg/2 weeks)	952	227
≤ 15	82 (8.6)	7 (3.1)
> 15 - 45	353 (37.1)	41 (18.1)
> 45 - 90	511 (53.7)	146 (64.3)
> 90 - 150	5 (0.5)	28 (12.3)
> 150	1 (0.1)	5 (2.2)
Mean (SD)	45.4 (30.77)	64.0 (32.85)

Note: Intact spleen at baseline was an inclusion criterion. BMD: bone mineral density; BMI: body mass index; EMEA: Europe, Middle East, Eurasia, and Africa; LATAM: Latin America (South America, Central America, and the Caribbean); JAPAC: Japan, Pacific and Asia; MN: multiples of normal

*Human Genome Variation Society (HGVS) standard nomenclature p.Leu483Pro (L483P) and p.Asn409Ser (N409S) are historically known as L444P and N370S, respectively

†For bone mineral density, the “baseline” assessment is the earliest measurement recorded after at least 4 years of treatment with ERT

^#^N for each parameter is provided above with the mean/median values for each parameter

### Hematologic parameters

**Fig. 2:**
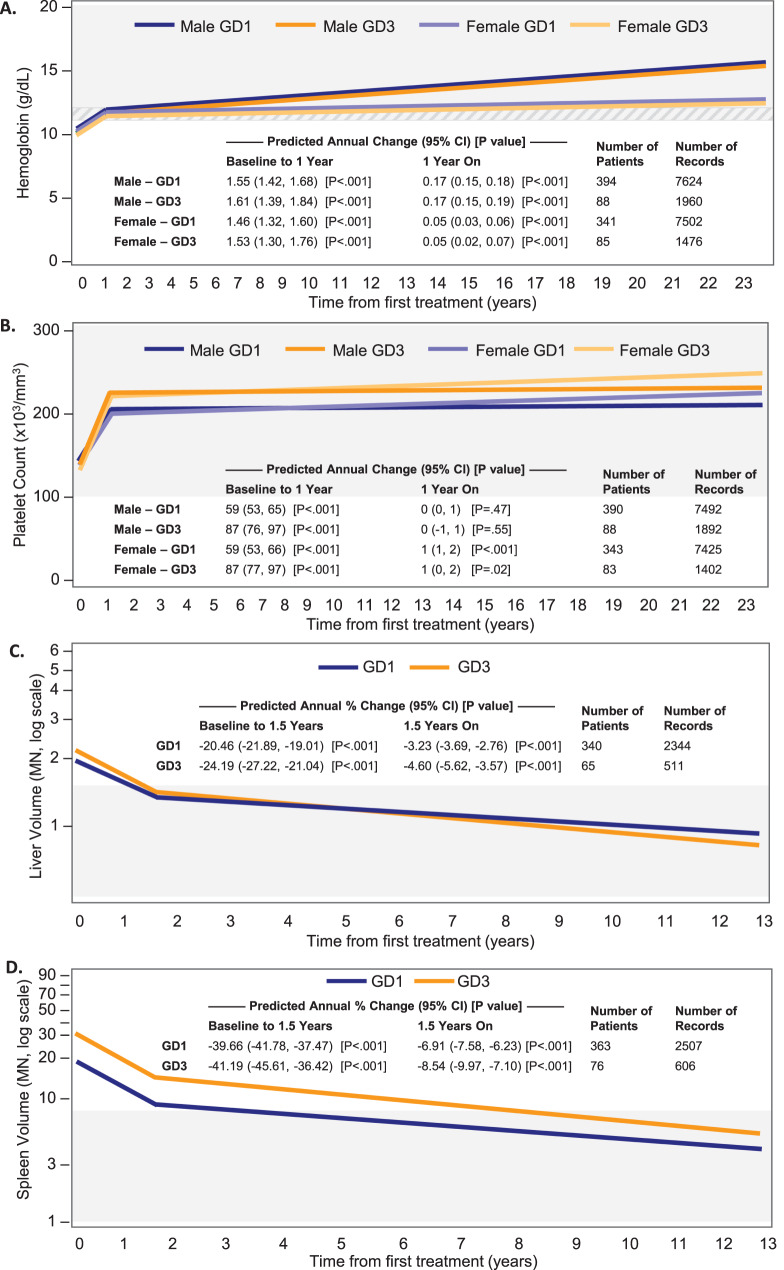
Estimated annual change (95% CI) following imiglucerase initiation in patients with GD1 (blue lines) and GD3 (orange lines). (**A**) Hemoglobin, (**B**) Platelet count, (**C**) Liver volume and (**D**) Spleen volume. Estimated values are provided by sex for parameters with interaction terms for sex and time included in the model (i.e., hemoglobin and platelet count). Liver and spleen volume data are log-transformed. Shaded areas represent long-term therapeutic goal thresholds for treated patients. [21, 22] Cross-hatched area represents difference between goal values for females (≥ 11.0 g/dL) and males (≥ 12.0 g/dL) >12 years of age

For hematologic parameters, children with GD1 and GD3 were followed for a median (25^th^, 75^th^ percentile) of 10.5 (4.9, 16.3) and 9.4 (4.0, 15.1) years, respectively, and contributed a median of 17 and 15 measurements per parameter, respectively. In the first year of imiglucerase therapy, hemoglobin and platelet counts showed statistically significant improvements across all groups. Hemoglobin increased an average of 1.46 g/dL (95% CI: 1.32, 1.60) per year among females with GD1, 1.55 g/dL (95% CI: 1.42, 1.68) among males with GD1, 1.53 g/dL (95% CI: 1.30, 1.76) among females with GD3, and 1.61 g/dL (95% CI: 1.39, 1.84) among males with GD3 (*p* < 0.001 for all, Fig. 2A). Platelet count increased an average of 59 × 10^3^/mm^3^ (95% CI: 53, 66) per year among females with GD1, 59 × 10^3^/mm^3^ (95% CI: 53, 65) among males with GD1, 87 × 10^3^/mm^3^ (95% CI: 77, 97) among females with GD3, and 87 × 10^3^/mm^3^ (95% CI: 76, 97) among males with GD3 (*p* < 0.001 for all, Fig. 2B). From year 1 to year 23, smaller average annual increases were observed for hemoglobin in all sex-phenotype combinations (*p* < 0.001 for all, Fig. 2A) and for platelet count in females of both phenotypes (*p* < 0.001 GD1, *p* = 0.02 GD3). Platelet counts remained stable among males with both GD1 and GD3 (Fig. 2B).

### Organ volumes

For organ volumes, children with GD1 and GD3 were followed for a median (25^th^, 75^th^ percentile) of 7.5 (3.5, 11.4) and 7.5 (4.2, 11.1) years, respectively, and contributed a median of 6 and 7 measurements per parameter, respectively. During the first 1.5 years of imiglucerase therapy, liver volume decreased an average of 20.46% per year (95% CI: −21.89, −19.01) in children with GD1 and 24.19% per year (95% CI: −27.22, −21.04) in children with GD3 (*p* < 0.001 for both, Fig. 2C); spleen volume decreased an average of 39.66% per year (95% CI: −41.78, −37.47) in children with GD1 and 41.19% per year (95% CI: −45.61, −36.42) in children with GD3 (*p* < 0.001 for both, Fig. 2D). From 1.5 to 13 years, organomegaly continued to improve, though at a slower pace (*p* < 0.001 for both, Fig. 2C and Fig. 2D).

### Growth

**Fig. 3:**
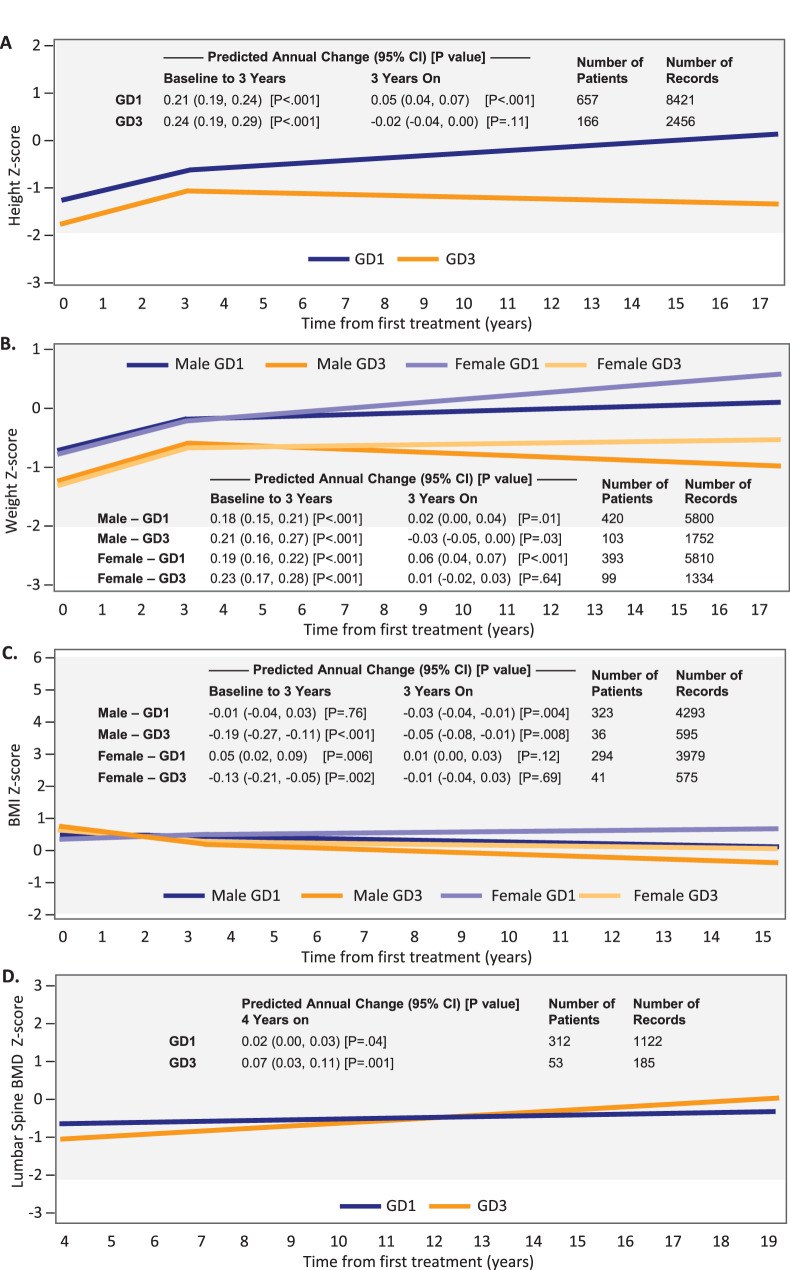
Estimated annual change (95% CI) in Z-scores for height (**A**), weight (**B**), BMI (**C**) and total lumbar spine BMD (**D**) following imiglucerase initiation in patients with GD1 (blue lines) and GD3 (orange lines). Estimated values are provided by sex for parameters with interaction terms included in the model (i.e., weight Z-score and BMI Z-score). Shaded areas represent long-term therapeutic goal thresholds for treated patients [21, 22]

Growth parameters were followed in children with GD1 and GD3 for a median (25^th^, 75^th^ percentile) of 7.1 (3.8, 10.8) and 9.4 (4.4, 13.5) years, respectively, with a median of 10–11 and 13–14 measurements per growth parameter, respectively. Baseline height and weight Z-scores in Table 1 reflect failure to thrive in the GD3 cohort and some children with GD1. Height Z-scores increased significantly during the first 3 years of imiglucerase therapy by an average of 0.21 (95% CI: 0.19, 0.24) per year in children with GD1 and 0.24 (95% CI: 0.19, 0.29) per year in children with GD3 (*p* < 0.001 for both, Fig. 3A). Weight Z-scores increased significantly during the first 3 years of imiglucerase therapy by an average of 0.19 (95% CI: 0.16, 0.22) per year in females with GD1, 0.18 (95% CI: 0.15, 0.21) in males with GD1, 0.23 (95% CI: 0.17, 0.28) in females with GD3, and 0.21 (95% CI: 0.16, 0.27) in males with GD3 (*p* < 0.001 for all, Fig. 3B). Between year 3 and year 17, height Z-scores continued to increase slightly each year in children with GD1 (*p* < 0.001) and remained stable in children with GD3 (Fig. 3A); weight Z-scores continued to increase slightly each year in children with GD1 (*p* < 0.001 for females, *p* = 0.01 for males), remained stable in females with GD3 and decreased slightly in males with GD3 (*p* = 0.03) (Fig. 3B). BMI Z-scores decreased or remained stable in patients with GD1 and GD3 (Fig. 3C). Estimated mean height and weight Z-scores at age 18 years were in the normal reference ranges for children with both GD1 and GD3 (Additional file 1), reflecting catch-up growth during time on treatment.

### Bone density

For total lumbar spine BMD Z-score, children with GD1 and GD3 were followed for a median (25^th^, 75^th^ percentile) of 9.7 (6.4, 12.8) and 9.9 (5.4, 13.8) years, respectively, and contributed a median of three measurements. Total lumbar spine BMD Z-scores at baseline were low (mean −0.6) in both cohorts (Table 1), however, this was based on measurements taken at four years on treatment due to the lack of feasibility in performing DXA in even smaller children. Beginning 4 years after initiating imiglucerase treatment and continuing through year 19, total lumbar spine BMD Z-score increased an average of 0.02 (95% CI: 0.00, 0.03) per year in children with GD1 (*p* = 0.04) and 0.07 (95% CI: 0.03, 0.11) per year in children with GD3 (*p* = 0.001) (Fig. 3D). At age 18 years, predicted mean total lumbar spine BMD Z-score was −0.42 (95% CI: −0.68, −0.15) in males with GD1, −0.31 (95% CI −0.57, −0.06) in females with GD1, −0.18 (95% CI: −0.58, 0.22) in males with GD3, and −0.08 (95% CI: −0.49, 0.33) in females with GD3 (Additional file 1).

### Sensitivity analysis

Because children with GD3 began imiglucerase treatment at a younger age (median 1.9 years) than those with GD1 (median 7.8 years), we conducted a sensitivity analysis focusing on children who started treatment before age six. The findings were consistent with the overall results (Additional file 2).

### Therapeutic goals

**Fig. 4:**
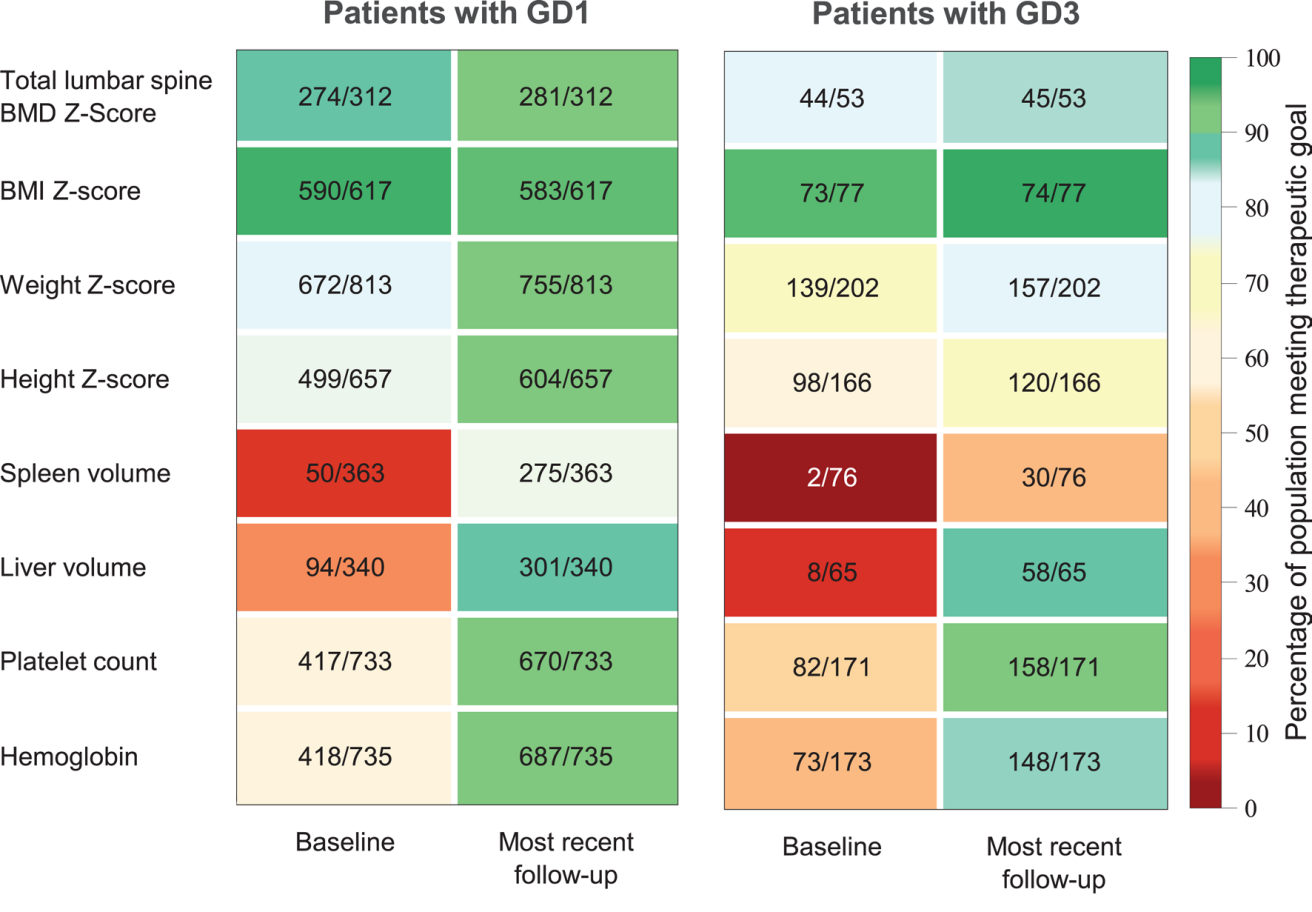
Heatmaps showing percentage of children meeting therapeutic goals at baseline and most recent assessment following imiglucerase initiation among Registry patients with GD1 and patients with GD3. Colors represent the percentage of patients meeting the therapeutic goal for each parameter, ranging from dark red (lowest percentage) to dark green (highest percentage). Therapeutic goals for patients with GD on enzyme replacement therapy: hemoglobin concentration ≥10.1 g/dL for infants < 6 months of age, ≥9.5 g/dL for children ≥6 months to ≤ 2 years of age, ≥10.5 g/dL for children > 2 to ≤ 12 years of age, ≥11.0 g/dL for females > 12 years of age and ≥12.0 g/dL for males > 12 years of age; platelet count ≥100 × 10^3^/mm^3^; spleen volume ≤8 MN; liver volume ≤1.5 MN; height, weight, BMI, and total lumbar spine BMD Z-scores > −2. [21, 22] Numbers in each box represent the number of patients achieving the goal out of the total number of patients in the cohort who had each assessment (n/N). Note: most recent assessment may be in adulthood. BMD: bone mineral density; BMI: body mass index

The percentage of children with GD1 and GD3 achieving therapeutic goals for hematologic, organ volume, growth (except BMI in GD1), and skeletal parameters increased numerically from baseline to most recent follow-up (Fig. 4 and Additional file 3) and from baseline to age 18 years (Additional file 4). Estimated mean values for all clinical parameters at age 18 years were within therapeutic goal ranges for patients with GD1 and GD3 treated with imiglucerase (Additional file 1), and estimated mean values for BMI Z-scores were below cutoffs for overweight or obesity.

## Discussion

This study represents the most comprehensive global analysis to date on the long-term impact of imiglucerase therapy in pediatric GD. Leveraging the largest international cohort of children with GD1 and GD3, our findings provide unequivocal evidence that imiglucerase is highly effective in reversing severe hematovisceral burden and promoting sustained improvements in growth and bone health, even in the most profoundly affected children with GD. Despite this extraordinary disease burden, imiglucerase therapy led to rapid and marked improvements, particularly in hematologic and visceral parameters, within the first one to two years of treatment. These benefits persisted over time, with statistically significant annual gains observed in BMD, organ volume reduction, and catch-up growth. Importantly, hematovisceral, skeletal, and most growth parameters—key indicators of disease severity in GD—were maintained within or moved into widely accepted therapeutic goal ranges during more than a decade of treatment. These findings highlight the rapid and sustained disease reversal in children with GD3, closely mirroring the long-term therapeutic outcomes observed in GD1.

Total lumbar spine BMD assessed starting four years after imiglucerase initiation showed consistent annual improvement. At imiglucerase initiation, most children were below the typical age for routine BMD assessments, leading to limited data. In a study where BMD was measured in very young children, osteopenia reversed rapidly with imiglucerase treatment [23]. Our BMD data collected at four years after starting imiglucerase likely reflects early treatment response, yet BMD remained low across the cohort, with children with GD3 more severely affected. Total lumbar spine BMD Z-scores increased significantly in children with GD1 and GD3 such that, by age 18 years, the predicted mean total lumbar spine BMD Z-score reflected near normal peak bone mass. Previous studies have highlighted an underlying osteoblastic defect in GD, leading to impaired bone formation and failure to attain peak bone mass without treatment—placing individuals on a lifelong trajectory toward osteoporosis [23, 26]. The impact of imiglucerase therapy on bone health of children with GD1 and GD3 helps mitigate these skeletal deficits and promotes BMD normalization. However, the true extent of its benefits may be underestimated, as early improvements within the first four years of therapy were not captured in our analysis.

The heatmaps (Fig. 4) illustrate substantial improvements with imiglucerase therapy in both GD1 and GD3, despite the overwhelming baseline disease burden in GD3. At treatment initiation, far fewer patients with GD3 than GD1 met therapeutic targets, reflecting profound hematologic and visceral involvement, including massive organomegaly and severe failure to thrive. While therapeutic goals were developed for GD1 and may not fully capture the complexities of GD3, the data demonstrate marked improvements with imiglucerase therapy across both groups, and a significant proportion of patients with GD3 moving closer to or achieving these predefined targets over time. Importantly, the response to imiglucerase in GD3 was transformative and potentially life-saving [12], as evidenced by the extreme baseline disease burden in these infants. Despite not always reaching conventional therapeutic goals, the improvements in hematologic and visceral parameters were substantial, and the reversal of massive organomegaly and critical cytopenias likely impacted survival. While skeletal and platelet count improvements were more gradual, the overall trajectory was positive. These findings reinforce the critical importance of early intervention, as newborn screening and prompt therapy initiation have the potential to prevent irreversible complications, reshape disease progression, and dramatically improve long-term health outcomes in children with GD3.

A normal growth trajectory is a fundamental indicator of overall health and well-being in children, and its disruption in GD reflects significant disease burden. At baseline, children with GD exhibited marked growth failure. Imiglucerase therapy led to substantial catch-up growth during the first three years of treatment; however, in GD3, growth trajectories eventually plateaued and therapeutic growth targets were not met by most patients. This flattening is likely attributable to greater disease severity in GD3, compounded by higher prevalence of kyphoscoliosis and other skeletal complications compared to GD1 [5]. In GD3, spinal deformities typically emerge during the second decade of life, exerting a profound impact on height and long-term growth potential [5, 27, 28]. We speculate that persistent skeletal abnormalities, rather than lack of systemic disease control, contributed to growth limitations in children with GD3, but the Registry does not systematically document skeletal deformities. More comprehensive musculoskeletal assessments in children with GD3 are needed to better understand long-term treatment efficacy and optimize patient care.

Differentiating between GD1 and GD3 in very young children is complicated by the broad phenotypic spectrum and variable onset of neurological symptoms, which may emerge anytime from infancy to young adulthood [3, 29]. Children with GD3 may initially be misclassified as having GD1 if neurological manifestations have not yet emerged or are too subtle to detect early. This is underscored by the presence of the classic neuronopathic GD (p.Leu483Pro/p.Leu483Pro) genotype in 32 (4.0%) children with GD1 compared to 141 (66.5%) children with GD3. Individuals harboring this genotype almost universally develop neurological symptoms at some point in their lives, which highlights the inherent challenges in early classification and the potential for delayed recognition of neuronopathic disease. In the Registry, GD classification was determined by the treating physicians and may have been misclassified in a small subset of patients, particularly in younger patients where the full disease trajectory is not yet apparent.

Neurologic disease is a defining feature of GD3, yet effective treatment remains elusive [4–6]. Although macrophage-targeted ERTs like imiglucerase have traditionally been considered incapable of crossing the blood-brain barrier [30], this assertion is largely based on assumption rather than direct evidence. Some clinical reports have documented stabilization or improvement of neurological symptoms in ERT-treated patients with GD3, suggesting that its effects on the CNS may be more complex than previously thought [10, 17, 31–33]. While these perceived benefits may arise indirectly from reduction of the overwhelming systemic inflammation [34] associated with hematovisceral and skeletal burden, recent studies indicate that the blood-brain barrier in neuronopathic GD may exhibit transient permeability under certain conditions. Glucosylceramide-laden immune cells have been shown to infiltrate the brain, potentially amplifying neuroinflammation [2], and peripheral imiglucerase molecules have been detected within glial cells [35], suggesting some degree of CNS uptake. Long-term disease modification in GD3 will likely require brain-penetrant substrate reduction therapy for neurological disease as a complement to systemic ERT for peripheral disease.

The ICGG Gaucher Registry is a robust, worldwide, observational database that provides valuable real-world insights. However, voluntary data entry results in potential data gaps (particularly organ volume assessments), and the Registry does not collect safety data (which is tracked via worldwide pharmacovigilance surveillance networks [36–39]), total-body BMD (excluding the head), or key lifestyle factors, such as nutritional status and physical activity, which may influence bone health and growth. Analysis of the GD biomarker chitotriosidase was not feasible. *CHIT1* genotype information, which is required to interpret chitotriosidase activity due to a common *CHIT1* null allele polymorphism (i.e., no activity in homozygotes and half normal activity in heterozygotes), is not collected in the Registry. In worldwide registry populations, variability in how organ volumes and BMD are measured may result in random variability that would likely decrease precision. In our analysis, patients were compared to themselves, thereby eliminating potential confounding by factors that do not vary over time. Finally, the mean dose in these children was 45 IU/kg/2 weeks for GD1 and 64 IU/kg/2 weeks for GD3, which aligns with standard-of-care recommendations across global treatment centers. However, dose availability and prescribing practices vary globally and future analyses stratifying patients by dose could provide additional insights into optimal treatment strategies in different healthcare settings. Despite these limitations, the Registry remains the most comprehensive and long-standing source of real-world data on pediatric GD, providing valuable insights into long-term treatment outcomes. By capturing the extraordinary heterogeneity of GD across diverse genotypes, geographies, and healthcare systems, this study delivers critical evidence that controlled trials alone cannot provide. As global efforts continue to refine treatment strategies and expand access to care, the insights gained from this Registry will remain essential in shaping evidence-based guidelines and improving outcomes for children with GD worldwide.

## Conclusions

Imiglucerase ERT facilitated catch-up growth and drove a sustained and profound reversal of hematovisceral and skeletal disease in severely affected children with both GD1 and GD3. These transformative benefits continued to accrue for more than a decade, with key clinical parameters consistently maintained within therapeutic targets. Despite the overwhelming disease burden in GD3, early initiation of imiglucerase enabled remarkable systemic disease reversal, underscoring its life-saving potential. These findings reinforce the critical urgency of newborn screening, early diagnosis, and sustained treatment as essential strategies to reshape the disease trajectory, prevent irreversible complications, and dramatically improve long-term survival and quality of life for affected children worldwide.

## Electronic supplementary material

Below is the link to the electronic supplementary material.

Supplementary Material 1

Supplementary Material 2

Supplementary Material 3

Supplementary Material 4

## Data Availability

Qualified researchers may request access to patient-level data and related study documents. Patient-level data will be anonymized, and study documents will be redacted to protect the privacy of trial participants. Further details on Sanofi’s data sharing criteria, eligible studies, and process for requesting access can be found at: https://vivli.org/.
