# Supplementary material for: The global impact of imiglucerase therapy in children with Gaucher disease types 1 and 3: a real-world analysis from the International Collaborative Gaucher Group Gaucher Registry
Source: Orphanet J Rare Dis. 2026 Mar 11;21:123. doi: 10.1186/s13023-026-04282-w (PMC13045098; doi:10.1186/s13023-026-04282-w)
Supplement: Supplementary file 1 — Supplementary Material 1 [file 13023_2026_4282_MOESM1_ESM.docx]

Additional File 1. Estimated mean values at age 18 years in children who initiated imiglucerase treatment at age 2 years. Values for organ volume and BMI are extrapolated. Shaded areas represent long-term therapeutic goal thresholds for treated patients.^1,2^ MN: multiples of normal.

**
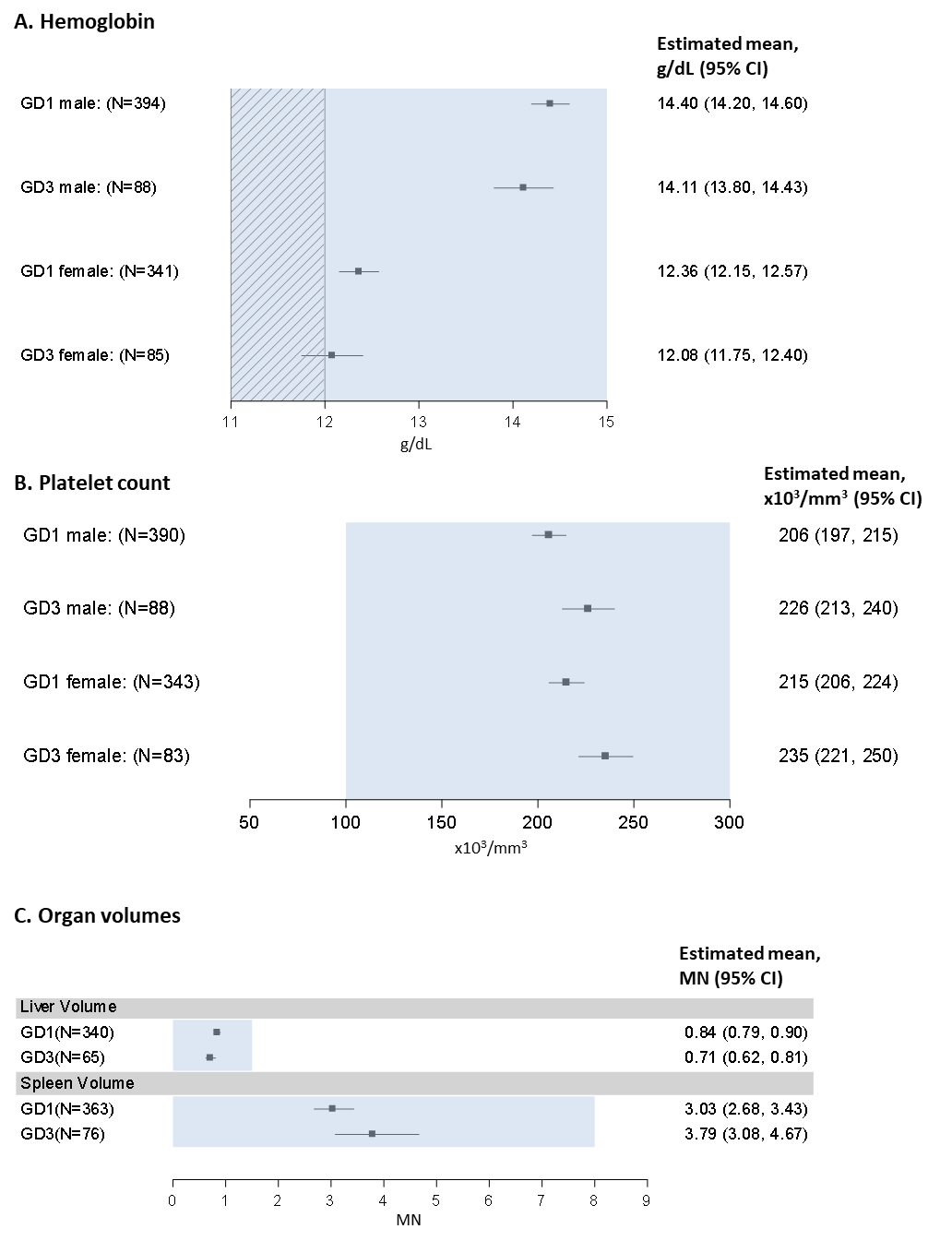
**

**
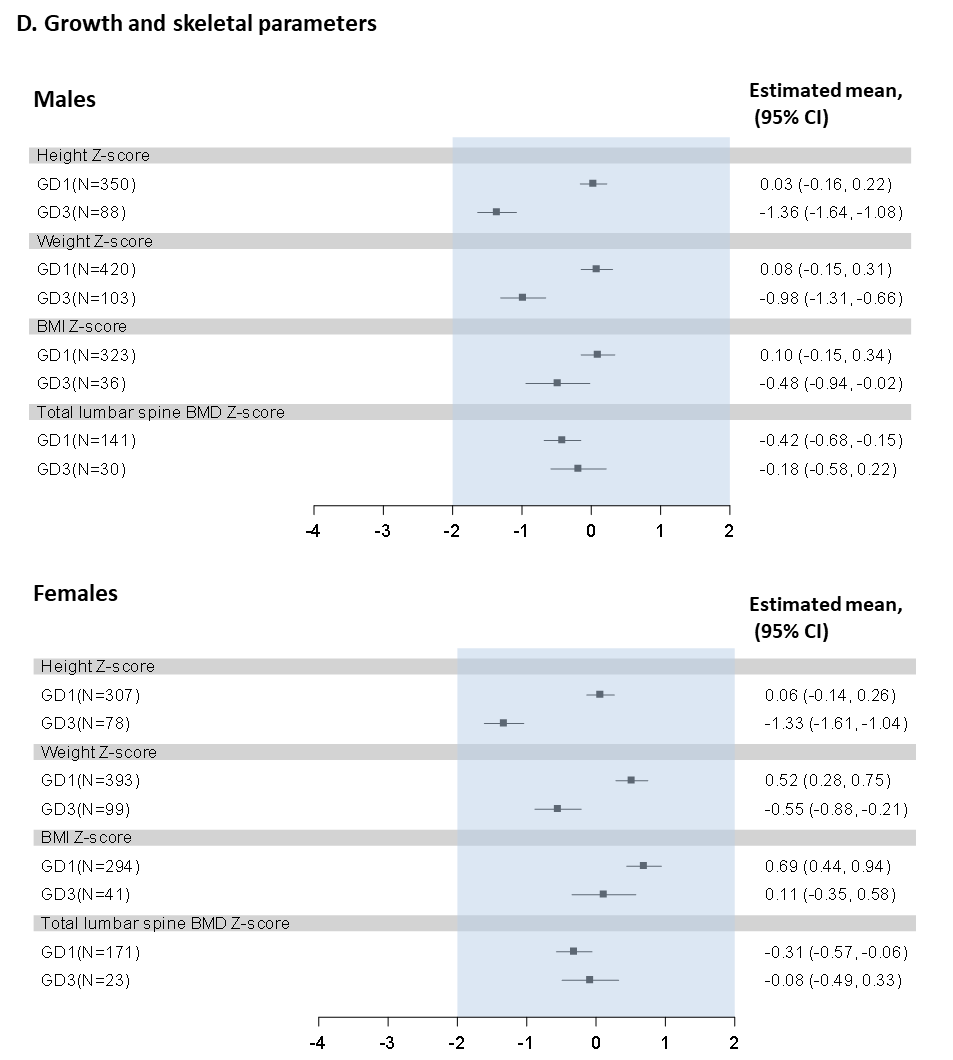
**

**References:**

1. Biegstraaten M, Cox TM, Belmatoug N, et al. Management goals for type 1 Gaucher disease: An expert consensus document from the European working group on Gaucher disease. *Blood Cells Mol Dis*. Feb 2018;68:203-208. doi:10.1016/j.bcmd.2016.10.008
2. Pastores GM, Weinreb NJ, Aerts H, et al. Therapeutic goals in the treatment of Gaucher disease. *Semin Hematol*. 2004;41(4 suppl 5):4-14. doi:S0037196304001325
