# Supplementary material for: The global impact of imiglucerase therapy in children with Gaucher disease types 1 and 3: a real-world analysis from the International Collaborative Gaucher Group Gaucher Registry
Source: Orphanet J Rare Dis. 2026 Mar 11;21:123. doi: 10.1186/s13023-026-04282-w (PMC13045098; doi:10.1186/s13023-026-04282-w)
Supplement: Supplementary file 2 — Supplementary Material 2 [file 13023_2026_4282_MOESM2_ESM.docx]

Additional File 2. Estimated annual change in each parameter following treatment initiation in sensitivity analysis of patients <6 years of age at first treatment

| **Hemoglobin** |  |  |  |  |  |  |
| --- | --- | --- | --- | --- | --- | --- |
| **Parameter** | **N Patients** | **N Records** | **Slope  Estimate** | **95% CI Lower  Limit** | **95% CI Upper  Limit** | **P Value** |
| Estimated annual change in hemoglobin (g/dL)^1^ in each time period | 433 | 8150 | - | - | - | - |
| Patients with GD1^2^ | 285 | 5307 | - | - | - | - |
| Males | 166 | 3043 | - | - | - | - |
| Baseline to 1 year^3^ | - | - | 1.48 | 1.28 | 1.67 | <0.001 |
| 1 year on | - | - | 0.18 | 0.16 | 0.20 | <0.001 |
| Females | 119 | 2264 | - | - | - | - |
| Baseline to 1 year | - | - | 1.49 | 1.27 | 1.71 | <0.001 |
| 1 year on | - | - | 0.10 | 0.07 | 0.12 | <0.001 |
| Patients with GD3 | 148 | 2843 | - | - | - | - |
| Males | 81 | 1797 | - | - | - | - |
| Baseline to 1 year | - | - | 1.60 | 1.34 | 1.85 | <0.001 |
| 1 year on | - | - | 0.16 | 0.14 | 0.19 | <0.001 |
| Females | 67 | 1046 | - | - | - | - |
| Baseline to 1 year | - | - | 1.61 | 1.34 | 1.88 | <0.001 |
| 1 year on | - | - | 0.08 | 0.05 | 0.10 | <0.001 |
| ^1^ Annual change estimated from linear mixed model with a slope estimated for two time periods: from treatment initiation to 1 year post-treatment initiation and from 1 year post-treatment initiation to the end of follow-up (23 years from first treatment). Model is adjusted for sex, age at first treatment, and disease phenotype and includes an interaction term between time and disease phenotype to allow different slopes for patients with GD1 and GD3. The model also includes an interaction term between time and sex to allow different slopes for males and females; this interaction was included because it was statistically significant and improved model fit. Results are shown as annual absolute change in the value.  ^2^ Disease type is as reported by physician. Patients received imiglucerase as first primary Gaucher therapy and no other concurrent therapy.  ^3^ "Baseline" is defined as the data point closest to imiglucerase initiation using a window of no more than -3 months to +2 weeks (inclusive) from initiation of therapy. | | | | | | |

| **Platelet Count** |  |  |  |  |  |  |
| --- | --- | --- | --- | --- | --- | --- |
| **Parameter** | **N Patients** | **N Records** | **Slope  Estimate** | **95% CI Lower  Limit** | **95% CI Upper  Limit** | **P Value** |
| Estimated annual change in platelet count (x10^3^/mm^3^)^1^ in each time period | 431 | 7994 | - | - | - | - |
| Patients with GD1^2^ | 286 | 5273 | - | - | - | - |
| Males | 164 | 2989 | - | - | - | - |
| Baseline to 1 year^3^ | - | - | 77 | 67 | 88 | <0.001 |
| 1 year on | - | - | 0 | -1 | 1 | 0.82 |
| Females | 122 | 2284 | - | - | - | - |
| Baseline to 1 year | - | - | 73 | 62 | 85 | <0.001 |
| 1 year on | - | - | 1 | 0 | 2 | 0.07 |
| Patients with GD3 | 145 | 2721 | - | - | - | - |
| Males | 80 | 1728 | - | - | - | - |
| Baseline to 1 year | - | - | 99 | 86 | 112 | <0.001 |
| 1 year on | - | - | 0 | -1 | 1 | 0.90 |
| Females | 65 | 993 | - | - | - | - |
| Baseline to 1 year | - | - | 95 | 81 | 109 | <0.001 |
| 1 year on | - | - | 1 | 0 | 2 | 0.15 |
| ^1^ Annual change estimated from linear mixed model with a slope estimated for two time periods: from treatment initiation to 1 year post-treatment initiation and from 1 year post-treatment initiation to the end of follow-up (23 years from first treatment). Model is adjusted for age at first treatment and disease phenotype and includes an interaction term between time and disease phenotype to allow different slopes for patients with GD1 and GD3. The model also includes an interaction term between time and sex to allow different slopes for males and females; this interaction was included because it was statistically significant and improved model fit. Results are shown as annual absolute change in the value.  ^2^ Disease type is as reported by physician. Patients received imiglucerase as first primary Gaucher therapy and no other concurrent therapy.  ^3^ "Baseline" is defined as the data point closest to imiglucerase initiation using a window of no more than -3 months to +2 weeks (inclusive) from initiation of therapy. | | | | | | |

| **Liver Volume** |  |  |  |  |  |  |
| --- | --- | --- | --- | --- | --- | --- |
| **Parameter** | **N Patients** | **N Records** | **Slope  Estimate** | **95% CI Lower  Limit** | **95% CI Upper  Limit** | **P Value** |
| Estimated annual % change in liver volume (MN)^1^ in each time period | 181 | 1368 | - | - | - | - |
| Patients with GD1^2^ | 125 | 933 | - | - | - | - |
| Baseline to 1.5 years^3^ | - | - | -20.70 | -23.09 | -18.24 | <0.001 |
| 1.5 years on | - | - | -4.23 | -4.93 | -3.52 | <0.001 |
| Patients with GD3 | 56 | 435 | - | - | - | - |
| Baseline to 1.5 years | - | - | -25.03 | -28.35 | -21.56 | <0.001 |
| 1.5 years on | - | - | -4.45 | -5.50 | -3.40 | <0.001 |
| ^1^ Annual change estimated from linear mixed model with a slope estimated for two time periods: from treatment initiation to 1.5 years post-treatment initiation and from 1.5 years post-treatment initiation to the end of follow-up (13 years from first treatment). Model is adjusted for age at first treatment and disease phenotype and includes an interaction term between time and disease phenotype to allow different slopes for patients with GD1 and GD3. Models for organ volume use log-transformed multiples of normal, and results are shown as annual percent change in organ volume MN.  ^2^ Disease type is as reported by physician. Patients received imiglucerase as first primary Gaucher therapy and no other concurrent therapy.  ^3^ "Baseline" is defined as the data point closest to imiglucerase initiation using a window of no more than -6 months to +6 weeks (inclusive) from initiation of therapy. | | | | | | |

| **Spleen Volume** |  |  |  |  |  |  |
| --- | --- | --- | --- | --- | --- | --- |
| **Parameter** | **N Patients** | **N Records** | **Slope  Estimate** | **95% CI Lower  Limit** | **95% CI Upper  Limit** | **P Value** |
| Estimated annual % change in spleen volume (MN)^1^ in each time period | 198 | 1484 | - | - | - | - |
| Patients with GD1^2^ | 131 | 957 | - | - | - | - |
| Baseline to 1.5 years^3^ | - | - | -39.12 | -43.09 | -34.88 | <0.001 |
| 1.5 years on | - | - | -7.64 | -8.66 | -6.60 | <0.001 |
| Patients with GD3 | 67 | 527 | - | - | - | - |
| Baseline to 1.5 years | - | - | -43.21 | -48.31 | -37.61 | <0.001 |
| 1.5 Years on | - | - | -8.16 | -9.60 | -6.70 | <0.001 |
| ^1^ Annual change estimated from linear mixed model with a slope estimated for two time periods: from treatment initiation to 1.5 years post-treatment initiation and from 1.5 years post-treatment initiation to the end of follow-up (13 years from first treatment). Model is adjusted for age at first treatment and disease phenotype and includes an interaction term between time and disease phenotype to allow different slopes for patients with GD1 and GD3. Models for organ volume use log-transformed multiples of normal, and results are shown as annual percent change in organ volume MN.  ^2^ Disease type is as reported by physician. Patients received imiglucerase as first primary Gaucher therapy and no other concurrent therapy.  ^3^ "Baseline" is defined as the data point closest to imiglucerase initiation using a window of no more than -6 months to +6 weeks (inclusive) from initiation of therapy. | | | | | | |

| **Height Z-score** |  |  |  |  |  |  |
| --- | --- | --- | --- | --- | --- | --- |
| **Parameter** | **N Patients** | **N Records** | **Slope  Estimate** | **95% CI Lower  Limit** | **95% CI Upper  Limit** | **P Value** |
| Estimated annual change in height Z-score^1^ in each time period | 416 | 6020 | - | - | - | - |
| Patients with GD1^2^ | 268 | 3754 | - | - | - | - |
| Baseline to 3 years^3^ | - | - | 0.16 | 0.12 | 0.20 | <0.001 |
| 3 Years on | - | - | 0.04 | 0.02 | 0.05 | <0.001 |
| Patients with GD3 | 148 | 2266 | - | - | - | - |
| Baseline to 3 years | - | - | 0.24 | 0.18 | 0.29 | <0.001 |
| 3 Years on | - | - | -0.02 | -0.04 | 0.00 | 0.05 |
| ^1^ Annual change estimated from linear mixed model with a slope estimated for two time periods: from treatment initiation to 3 years post-treatment initiation and from 3 years post-treatment initiation to the end of follow-up (17 years from first treatment). Model is adjusted for sex, age at first treatment, and disease phenotype and includes an interaction term between time and disease phenotype to allow different slopes for patients with GD1 and GD3. Results are shown as annual absolute change.  ^2^ Disease type is as reported by physician. Patients received imiglucerase as first primary Gaucher therapy and no other concurrent therapy.  ^3^ "Baseline" is defined as the data point closest to imiglucerase initiation using a window of no more than -3 months to +3 months (inclusive) from initiation of therapy. | | | | | | |

| **Weight Z-score** |  |  |  |  |  |  |
| --- | --- | --- | --- | --- | --- | --- |
| **Parameter** | **N Patients** | **N Records** | **Slope  Estimate** | **95% CI Lower  Limit** | **95% CI Upper  Limit** | **P Value** |
| Estimated annual change in weight Z-score^1^ in each time period | 492 | 7746 | - | - | - | - |
| Patients with GD1 ^2^ | 321 | 5009 | - | - | - | - |
| Males | 173 | 2561 | - | - | - | - |
| Baseline to 3 years^3^ | - | - | 0.17 | 0.12 | 0.22 | <0.001 |
| 3 years on | - | - | 0.01 | -0.01 | 0.03 | 0.26 |
| Females | 148 | 2448 | - | - | - | - |
| Baseline to 3 years | - | - | 0.13 | 0.08 | 0.18 | <0.001 |
| 3 years on | - | - | 0.04 | 0.01 | 0.06 | 0.002 |
| Patient with GD3 | 171 | 2737 | - | - | - | - |
| Males | 94 | 1636 | - | - | - | - |
| Baseline to 3 years | - | - | 0.24 | 0.18 | 0.30 | <0.001 |
| 3 years on | - | - | -0.03 | -0.06 | 0.00 | 0.03 |
| Females | 77 | 1101 | - | - | - | - |
| Baseline to 3 years | - | - | 0.20 | 0.13 | 0.26 | <0.001 |
| 3 Years on | - | - | -0.01 | -0.04 | 0.02 | 0.67 |
| ^1^ Annual change estimated from linear mixed model with a slope estimated for two time periods: from treatment initiation to 3 years post-treatment initiation and from 3 years post-treatment initiation to the end of follow-up (17 years from treatment initiation). Model is adjusted for sex, age at first treatment, and disease phenotype and includes an interaction term between time and disease phenotype to allow different slopes for GD1 and GD3 patients. The model also includes an interaction term between time and sex to allow different slopes for males and females; this interaction was included because it was statistically significant and improved model fit. Results are shown as annual absolute change.  ^2^ Disease type is as reported by physician. Patients received imiglucerase as first primary Gaucher therapy and no other concurrent therapy.  ^3^ "Baseline" is defined as the data point closest to imiglucerase initiation using a window of no more than -3 months to +3 months (inclusive) from initiation of therapy. | | | | | | |

| **BMI Z-score** |  |  |  |  |  |  |
| --- | --- | --- | --- | --- | --- | --- |
| **Parameter** | **N Patients** | **N Records** | **Slope  Estimate** | **95% CI Lower  Limit** | **95% CI Upper  Limit** | **P Value** |
| Estimated annual change in BMI Z-score^1^ in each time period | 280 | 4183 | - | - | - | - |
| Patients with GD1^2^ | 222 | 3234 | - | - | - | - |
| Males | 122 | 1780 | - | - | - | - |
| Baseline to 3 years^3^ | - | - | -0.06 | -0.12 | 0.00 | 0.05 |
| 3 years on | - | - | -0.03 | -0.05 | 0.00 | 0.03 |
| Females | 100 | 1454 | - | - | - | - |
| Baseline to 3 years | - | - | 0.01 | -0.06 | 0.08 | 0.80 |
| 3 years on | - | - | -0.01 | -0.04 | 0.02 | 0.41 |
| Patients with GD3 | 58 | 949 | - | - | - | - |
| Males | 29 | 514 | - | - | - | - |
| Baseline to 3 years | - | - | -0.23 | -0.33 | -0.13 | <0.001 |
| 3 years on | - | - | -0.04 | -0.08 | 0.00 | 0.07 |
| Females | 29 | 435 | - | - | - | - |
| Baseline to 3 years | - | - | -0.16 | -0.26 | -0.05 | 0.003 |
| 3 years on | - | - | -0.02 | -0.06 | 0.02 | 0.31 |
| ^1^ Annual change estimated from linear mixed model with a slope estimated for two time periods: from treatment initiation to 3 years post-treatment initiation and from 3 years post-treatment initiation to the end of follow-up (15 years from treatment initiation). Model is adjusted for sex, age at first treatment, and disease phenotype and includes an interaction term between time and disease phenotype to allow different slopes for patients with GD1 and GD3. Model also include an interaction term between time and sex to allow different slopes for males and females; this interaction was included because it was statistically significant and improved model fit. Results are shown as annual absolute change.  ^2^ Disease type is as reported by physician. Patients received imiglucerase as first primary Gaucher therapy and no other concurrent therapy.  ^3^ "Baseline" is defined as the data point closest to imiglucerase initiation using a window of no more than -3 months to +3 months (inclusive) from initiation of therapy. | | | | | | |
