# Supplementary material for: The global impact of imiglucerase therapy in children with Gaucher disease types 1 and 3: a real-world analysis from the International Collaborative Gaucher Group Gaucher Registry
Source: Orphanet J Rare Dis. 2026 Mar 11;21:123. doi: 10.1186/s13023-026-04282-w (PMC13045098; doi:10.1186/s13023-026-04282-w)
Supplement: Supplementary file 3 — Supplementary Material 3 [file 13023_2026_4282_MOESM3_ESM.docx]

Additional File 3. Plots of individual patient outcomes for each clinical parameter in GD1 and GD3 patients. Shaded areas represent long-term therapeutic goal thresholds for treated patients.^1,2^ Cross-hatched area represents difference between goal values for females (≥11.0 g/dL) and males (≥12.0 g/dL) >12 years of age.


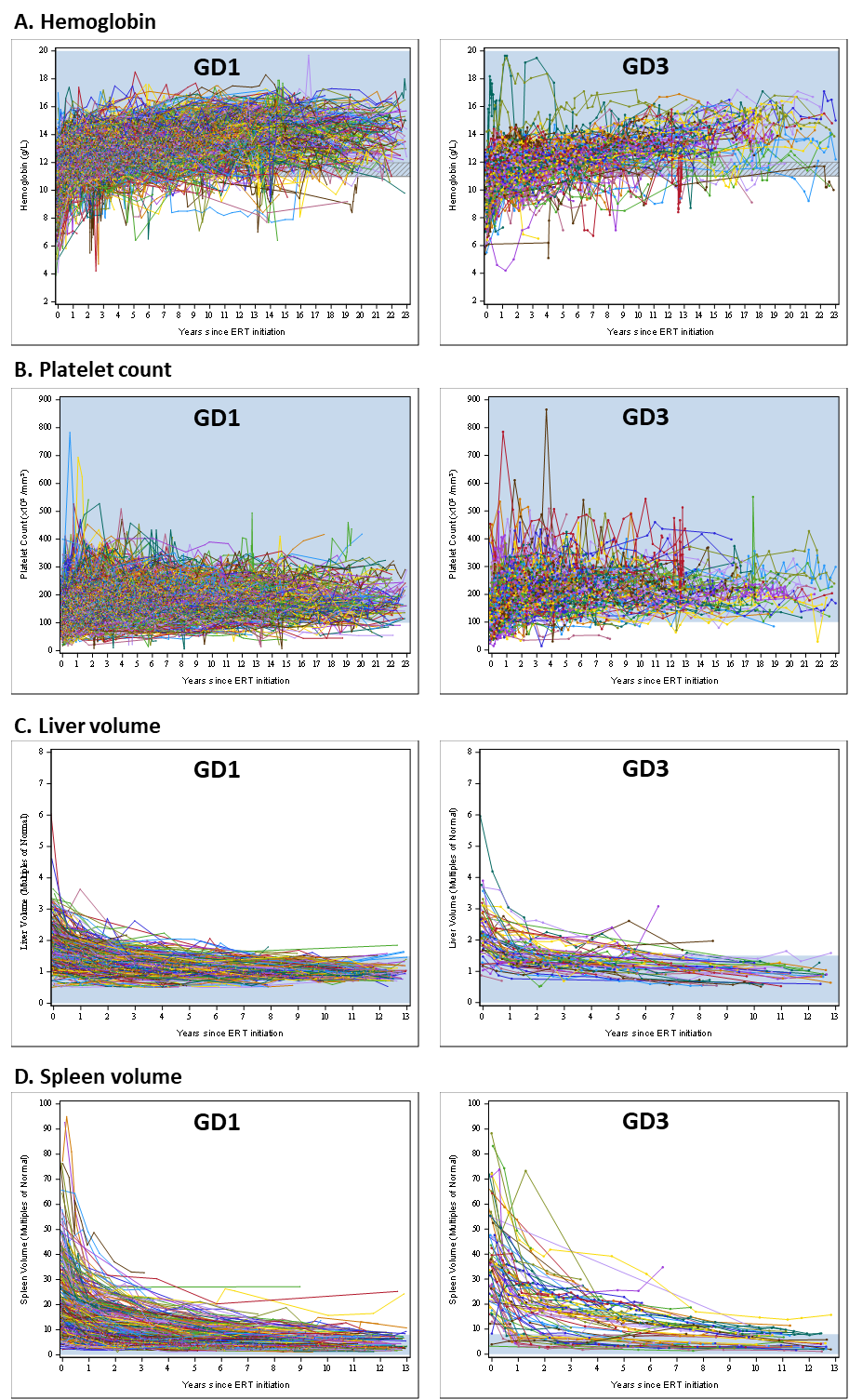


**
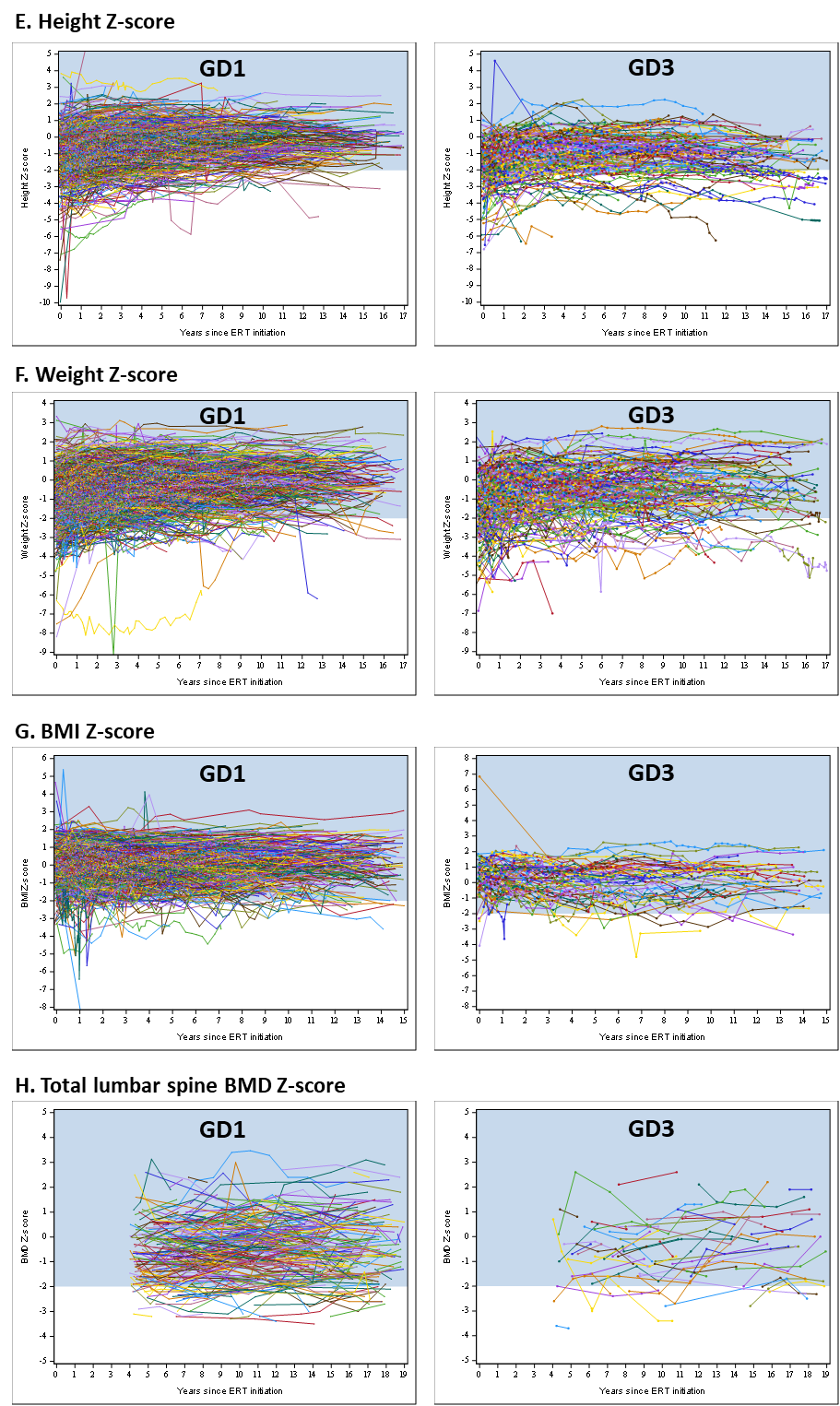
**

**References:**

1. Biegstraaten M, Cox TM, Belmatoug N, et al. Management goals for type 1 Gaucher disease: An expert consensus document from the European working group on Gaucher disease. *Blood Cells Mol Dis*. Feb 2018;68:203-208. doi:10.1016/j.bcmd.2016.10.008
2. Pastores GM, Weinreb NJ, Aerts H, et al. Therapeutic goals in the treatment of Gaucher disease. *Semin Hematol*. 2004;41(4 suppl 5):4-14. doi:S0037196304001325
