# Supplementary material for: The global impact of imiglucerase therapy in children with Gaucher disease types 1 and 3: a real-world analysis from the International Collaborative Gaucher Group Gaucher Registry
Source: Orphanet J Rare Dis. 2026 Mar 11;21:123. doi: 10.1186/s13023-026-04282-w (PMC13045098; doi:10.1186/s13023-026-04282-w)
Supplement: Supplementary file 4 — Supplementary Material 4 [file 13023_2026_4282_MOESM4_ESM.docx]

Additional File 4. Heatmaps showing percentage of children treated with imiglucerase who met therapeutic goals at baseline and at age 18 years among registry patients with GD1 and patients with GD3. Colors represent the percentage of patients meeting the therapeutic goal for each parameter, ranging from dark red (lowest percentage) to dark green (highest percentage). Therapeutic goals for GD patients on enzyme replacement therapy: hemoglobin concentration ≥10.1 g/dL for infants <6 months of age, ≥9.5 g/dL for children ≥6 months to ≤2 years of age, ≥10.5 g/dL for children >2 to ≤12 years of age, ≥11.0 g/dL for females >12 years of age and ≥12.0 g/dL for males >12 years of age; platelet count ≥100 x 10^3^/mm^3^; spleen volume ≤8 MN; liver volume ≤1.5 MN; height, weight, BMI, and total lumbar spine BMD Z-scores >-2.^1,2^ Numbers in each box represent the number of patients achieving the goal out of the total number of patients in the cohort who had each assessment (n/N). BMD: bone mineral density; BMI: body mass index.

**
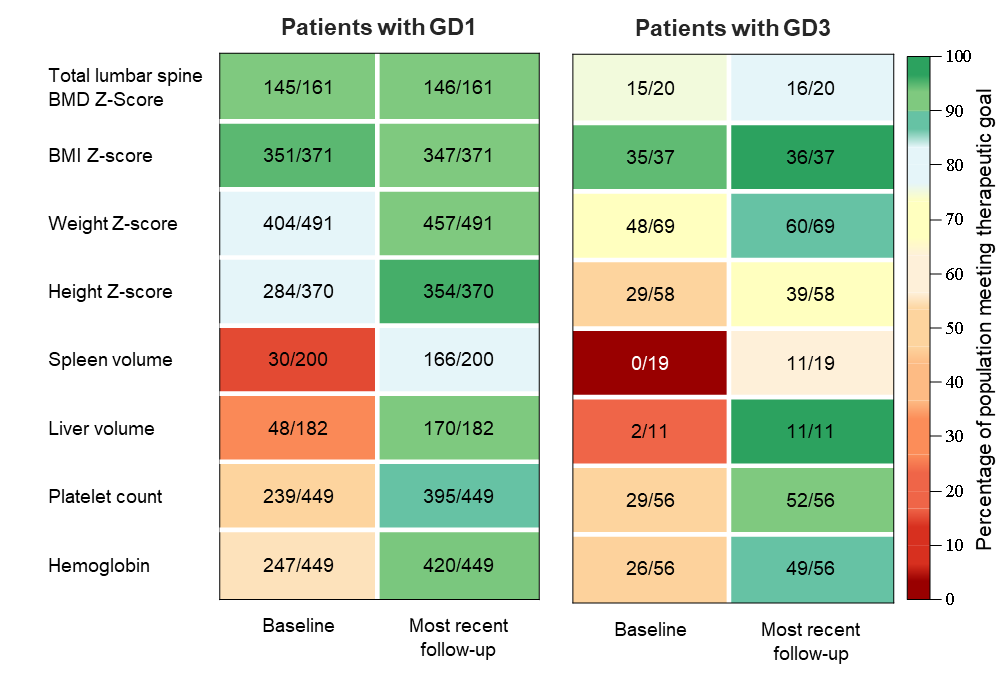
**

**References:**

1. Biegstraaten M, Cox TM, Belmatoug N, et al. Management goals for type 1 Gaucher disease: An expert consensus document from the European working group on Gaucher disease. *Blood Cells Mol Dis*. Feb 2018;68:203-208. doi:10.1016/j.bcmd.2016.10.008
2. Pastores GM, Weinreb NJ, Aerts H, et al. Therapeutic goals in the treatment of Gaucher disease. *Semin Hematol*. 2004;41(4 suppl 5):4-14. doi:S0037196304001325
